# A robust photoluminescence screening assay identifies uracil-DNA glycosylase inhibitors against prostate cancer†

**DOI:** 10.1039/c9sc05623h

**Published:** 2020-01-10

**Authors:** Guodong Li, Stuart Adam Henry, Hao Liu, Tian-Shu Kang, Sang-Cuo Nao, Yichao Zhao, Chun Wu, Jianwen Jin, Jia-Tong Zhang, Chung-Hang Leung, Philip Wai Hong Chan, Dik-Lung Ma

**Affiliations:** State Key Laboratory of Quality Research in Chinese Medicine, Institute of Chinese Medical Sciences, University of Macau Macau duncanleung@um.edu.mo; Department of Chemistry, University of Warwick Coventry CV4 7AL UK; Department of Chemistry, Hong Kong Baptist University Kowloon Tong Hong Kong edmondma@hkbu.edu.hk; School of Chemistry, Monash University Clayton Victoria 3800 Australia phil.chan@monash.edu

## Abstract

Many cancers have developed resistance to 5-FU, due to removal by the enzyme uracil-DNA glycosylase (UDG), a type of base excision repair enzyme (BER) that can excise uracil and 5-fluorouracil (5-FU) from DNA. However, the development of UDG inhibitor screening methods, especially for the rapid and efficient screening of natural product/natural product-like compounds, is still limited so far. We developed herein a robust time-resolved photoluminescence method for screening UDG inhibitors, which could significantly improve sensitivity over the screening method based on the conventional steady-state spectroscopy, reducing the substantial fluorescence background interference. As a proof-of-concept, two potential UDG inhibitors were identified from a database of natural products and approved drugs. Co-treatment of these two compounds with 5-FU showed synergistic cytotoxicity, providing the basis for treating drug-resistant cancers. Overall, this method provides an avenue for the rapid screening of small molecule regulators of other BER enzyme activities that can avoid false negatives arising from the background fluorescence.

## Abstract 2

The discovery of UDG inhibitors against prostate cancer by using a robust photoluminescence screening assay that can avoid false negatives arising from the background fluorescence.

## Graphical abstract

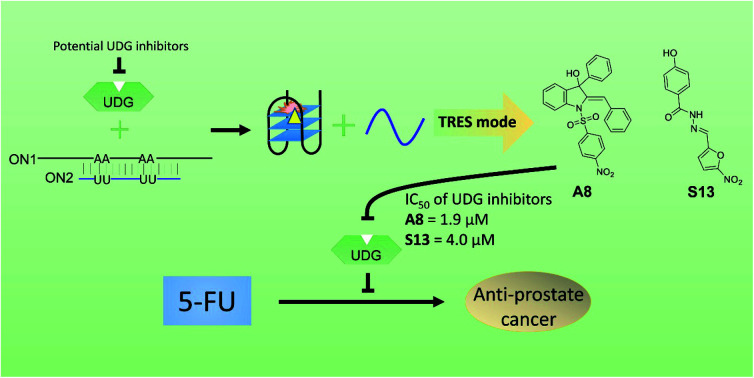

## Introduction

Prostate cancer is a malignancy that can remain latent for extended periods of time, resulting in a high disease burden.^1,2^ Prostate cancer ranks second among cancers in incidence among men, with 1 million new incidences of cancer reported every year.^3^ Radiotherapy, which involves using high frequency waves to destroy tumor cells,^4^ is commonly employed for prostate cancer treatment.^5^ However, radiotherapy exhibits certain adverse effects, including loss of appetite, vomiting, nausea, hair loss and sore skin.^6^ 5-Fluorouracil (5-FU), first patented in 1956 and entered into medical use in 1962,^7^ is effective against different cancers including prostate cancer through its ability to target thymidylate synthase, which leads to the incorporation of uracil and 5-FU into the genome.^8,9^ However, many cancers have also developed resistance to 5-FU, due to its removal from genomic DNA by the enzyme uracil-DNA glycosylase (UDG),^10–12^ a type of base excision repair enzyme (BER) that can excise uracil and 5-FU from DNA^13^ The depletion of UDG sensitizes tumor cells to 5-FU.^14^ The uracil-DNA glycosylase inhibitor (UDGI), produced in of *Bacillus subtilis* bacteriophage PBS1, is a ∼9.5 kDa protein that is used in the literature as a model inhibitor of UDG.^15^ Other inhibitors of UDG have been reported, such as SSP0047, p56, and uracil aldehyde small molecules,^13,16–20^ however none of these have undergone further in-depth disease application research. The discovery of new inhibitors of UDG and the development of methods for their identification could offer the potential for synergistic therapeutic strategies with 5-FU against cancer, including prostate cancer.

The combination of radioactive labeling with gel electrophoresis is deemed to be the “gold standard” for assaying DNA-modulating enzyme activity and for the identification of their modulators.^21^ Other reported methods for identifying UDG inhibitors include fragment-substrate tethering, bioinformatics, radioisotopic labeling, chemical cross-linking and affinity chromatography techniques.^13,16–20^ However, these methods tend to be time-consuming, unwieldy and/or may necessitate stringent safety measures to control radiographic exposure.^22^ Therefore, new *in vitro* strategies for the rapid and efficient screening of UDG inhibitors are still desired. In comparison, steady-state fluorescence spectroscopy^23,24^ has attracted interest as a tool to detect DNA repair enzyme activity, as optical strategies are more convenient and simpler.^25–27^ However, the use of oligonucleotides labeled with organic dyes is limited by the high cost of synthesis of labeled DNA.^28,29^ Moreover, the nanosecond lifetime of organic dyes is generally too short to allow their fluorescence to be separated from the high background fluorescence of samples and can result in false negatives, which greatly limits their drug screening applications.^30^ Consequently, the problem of background fluorescence is a major concern for the screening of small molecule inhibitors.^31^ Time-resolved emission spectroscopy (TRES) is a technique that measures the emission at discrete times during the fluorescence decay process,^32^ which provides an potential route to overcome short-lived fluorescence signals. Recently, TRES has been used to detect a variety of analytes, such as mercury ions, aluminum ions, and mRNA.^33–35^ However, reports describing the detection of enzymes with DNA-modulating activities by TRES are still limited.

**Fig. 1:**
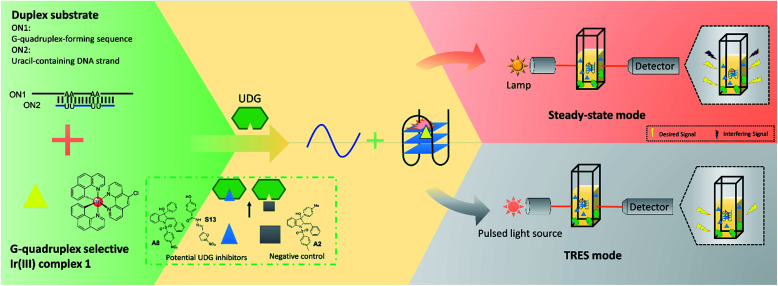
Schematic diagram of the novel UDG inhibitor screening method. In the assay mechanism, the G-quadruplex-forming motif (ON1, 5′-G_3_TAG_3_A_3_T_2_CT_2_A_2_GTGCG_3_T_2_G_3_-3′) is hybridized initially with a partly complementary, uracil-containing DNA sequence (ON2, 5′-CGCACU_2_A_2_GA_2_U_2_TC-3′) to form a double-stranded DNA substrate. The addition of UDG excises uracil bases from ON1–ON2, forming abasic sites on ON2 which releases ON1. ON1 converts into a G-quadruplex motif that is then bound by the iridium(iii) complex **1** with enhanced emission. In the presence of a UDG inhibitor, less ON1 would be liberated, resulting in a weaker luminescence intensity from probe **1**. However, this decrease might become completely swamped by the background fluorescence, resulting in a false negative result (upper panel). By using TRES, the short-lived fluorescence of inhibitors would be eliminated, allowing the reducing in luminescence intensity of probe **1** to be clearly detected (lower panel).

In this study, we developed a robust UDG inhibitor screening method through combining a G-quadruplex-specific long-lived luminescent iridium(iii) probe with a DNA-switching strategy and TRES. In this method, the G-quadruplex-forming sequence (ON1, 5′-G_3_TAG_3_A_3_T_2_CT_2_A_2_GTGCG_3_T_2_G_3_-3′) is initially hybridized with a partially complementary, uracil-containing DNA strand (ON2, 5′-CGCACU_2_A_2_GA_2_U_2_TC-3′) to form a duplex substrate (Fig. 1). Uracil, an undesired component of DNA that is produced from the hydrolysis of cytosine, is excised by UDG to form abasic sites.^36–38^ In our system, the presence of UDG is expected to create four abasic sites on ON2, which will greatly weaken the interaction between ON1 and ON2 due to the loss of four A–U complementary base pairs. This allows liberation of ON1, which is then able to fold into a G-quadruplex conformation that is subsequently recognized by the G-quadruplex-selective iridium(iii) complex with an enhanced luminescence response. However, if an UDG inhibitor is present, the release of ON1 will be prevented and thus the emission of the iridium(iii) complex will remain low. Our assay also exploits the long-lived phosphorescence emission and large Stoke shifts of the triplet-state energy levels of iridium(iii) complexes display,^39^ allowing their emission to be discriminated even in a highly fluorescent background.^40,41^

**Fig. 2:**
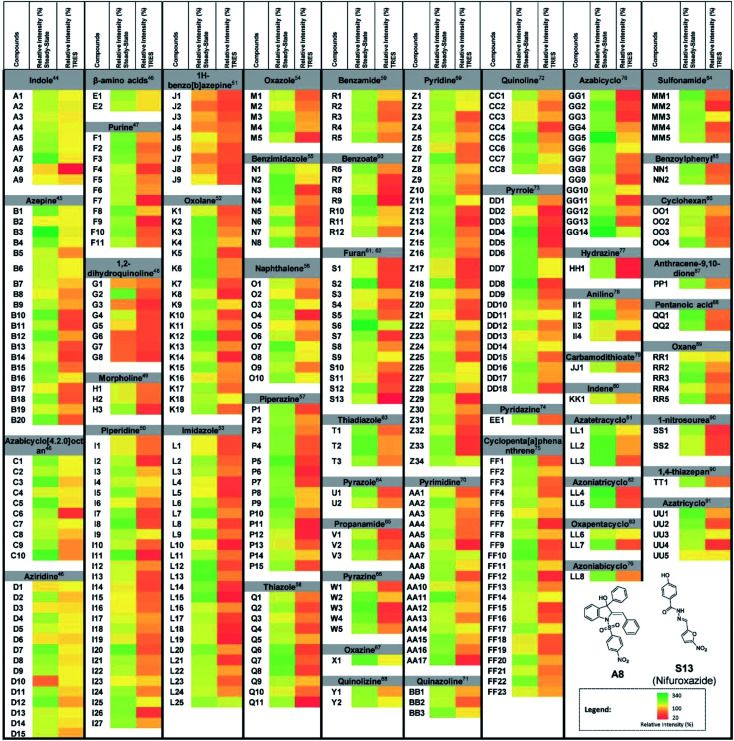
UDG inhibitor screening from a privileged library. Screening results for 400 natural product-like or FDA/EMA-approved compounds (10 μM) on UDG activity by the steady-state emission and TRES methods, with 100 U mL^−1^ UDGI as positive control. The duplex substrate was incubated with the indicated concentrations of UDG and 10 μM natural product-like compounds. The mixture was heated to 37 °C for 30 min to allow the base cleavage reaction to take place. The mixture was cooled down and was subsequently diluted using Tris buffer to a final volume of 500 μL. 0.5 μM of complex **1** was added to the mixture. **A8** and **S13** were identified as potential UDG inhibitors.

As a proof-of-concept, we used this screening method to identify potential UDG inhibitors from a privileged library of 400 natural product-like or FDA/EMA-approved compounds (Fig. 2). These compounds span an extensive array of potential bioactive chemical scaffolds that may be involved in regulating the DNA damage response or possess antitumor activity, including saturated and unsaturated heterocycles, β-amino acids, sulfonamides, carboxylic acids, among other classes. These compounds may generate a strong background fluorescence when screening in steady-state mode, which would interfere with the screening of UDG inhibitors. Therefore, we developed a TRES method to screen our library, which eventually led to the identification of the natural product-like indole derivative (**A8**) and an FDA-approved antibiotic drug nifuroxazide (**S13**), currently used against colitis and diarrhea, as inhibitors of UDG. In this study, we used our developed TRES screening method to identify a natural product-like indole derivative (**A8**) and an FDA-approved antibiotic drug nifuroxazide (**S13**), currently used against colitis and diarrhoea, as inhibitors of UDG. Both compounds also showed synergistic cytotoxicity with 5-FU, providing a basis for treating drug-resistant cancers, such as advanced prostate cancer. A schematic of the screening method for UDG inhibitors is presented in Fig. 1. We anticipate that this study could encourage the development of future TRES platforms for screening inhibitors of DNA repair enzymes, especially for molecules that are naturally fluorescent, enabling the rapid discovery of bioactive compounds from scaffolds that were previously neglected because of their high false positive rate.

## Results

### Development of TRES methodology for screening UDG inhibitors

To confirm the formation of the ON1 G-quadruplex structure, circular dichroism (CD) spectroscopy was performed. CD spectroscopy of the ON1 sequence showed a strong positive peak at 287 nm with a shoulder at 266 nm and a weaker negative peak at 245 nm, consistent with a hybrid-type G-quadruplex (Fig. S1†).^42,43^ We further evaluated the selectivity of **1** for the G-quadruplex DNA sequence. We found that **1** displayed significantly enhanced luminescence in the presence of G-quadruplex DNA (ON1 and c-kit87), while only slight luminescence changes were observed in the presence of single-stranded DNA or double-stranded DNA (Fig. S2†). These results demonstrate that complex **1** can selectively recognize the ON1 G-quadruplex leading to an enhanced luminescence response, and is consistent with previous reports.^38^

In steady-state mode, almost all of the test samples showed higher emission signals than the vehicle control (Fig. 2), indicating that the steady-state mode is highly susceptible false negatives arising from fluorescence interference. However, we determined that complex **1** had a luminescence lifetime of 931 ns, which was over nine times as long as the 400 natural product-like or FDA/EMA-approved compounds, which had lifetimes less than 100 ns. Therefore, we anticipated that the fluorescence of the samples could be avoided by screening the library under TRES conditions. As expected, the obvious false signals in steady-state mode could be effectively eliminated by using TRES mode (Fig. 2), demonstrating that the TRES-based screening method is a viable technique to screen libraries with a larger quantity and variety of scaffolds. Overall, these data suggest the TRES-based screening method that we developed in this work could effectively eliminate the problem of background fluorescence, allowing false negatives to be avoided.

**Fig. 3:**
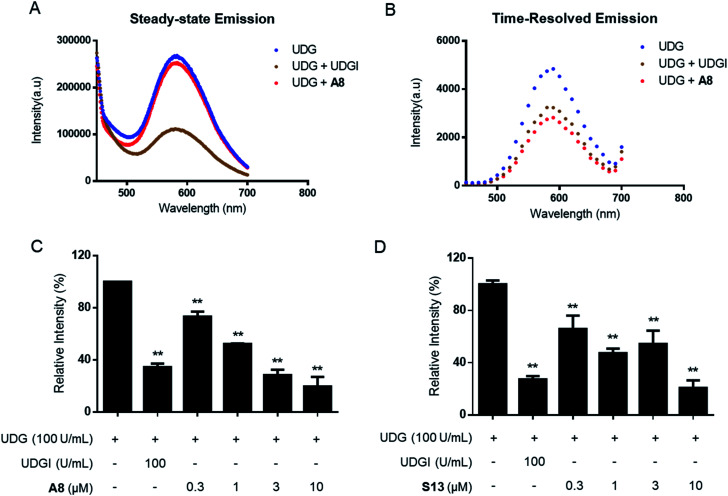
TRES-based screening method could effectively avoid false negatives. (A and B) G-quadruplex/**1** method in steady-state emission mode or TRES mode in the presence of 100 U mL^−1^ UDG, 100 U mL^−1^ UDG + 100 U mL^−1^ UDGI, or 100 U mL^−1^ UDG + 10 μM **A8**. Luminescence emission intensity was recorded at 450–700 nm with excitation at 355 nm. (C) Dose effect of **A8** on UDG activity as measured by TRES. (D) Dose effect of **S13** on UDG activity as measured by TRES. Error bars represent the standard deviations of the results from three independent experiments. *P* values were calculated using a two-sided *t*-test. *P < 0.05, **P < 0.01 *vs.* UDG group, respectively.

In the TRES screen, two potent UDG inhibitors, the natural product-like indole compound **A8** and the FDA/EMA-approved drug nifuroxazide (**S13**) were identified. **A8** showed the highest inhibition of UDG activity (80.7% at 10 μM), compared to 78.7% for **S13** and 65.6% for the positive control compound UDGI.^92^ Complex **1** benefited from a larger Stokes shifts and a longer emission lifetime than **A8** (Fig. S3A–D†). Hence, in TRES mode, the long-lived luminescence lifetime of complex **1** can be easily separated from the short-lived fluorescence of compound **A8** (Fig. S3E and F†). Importantly, although compounds **A8** and **S13** showed promising UDG inhibitory activity in TRES mode, they displayed no apparent activity under steady-state conditions, and thus would be registered as an apparent false negative arising from fluorescence interference. Under TRES conditions, the ability of **A8** to inhibit UDG activity could be clearly observed, as revealed by a marked decrease in the emission levels of probe **1** (Fig. 3A and B). **A8** showed an IC_50_ of 1.9 μM at inhibiting UDG activity, while **S13** showed an IC_50_ of *ca.* 4.0 μM at inhibiting UDG activity (Fig. 3C and D).

### Verification of UDG activity inhibition by UDG inhibitors by DNA polyacrylamide gel electrophoresis

**Fig. 4:**
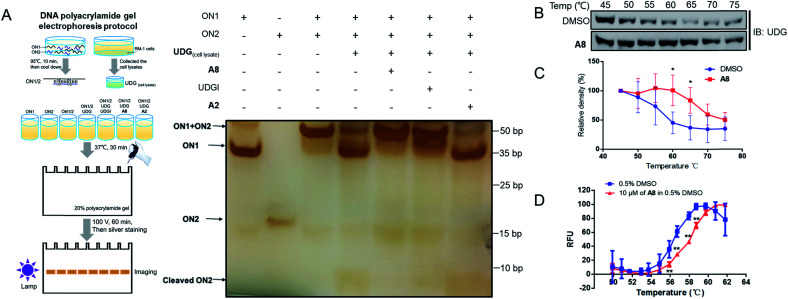
**A8** could engage UDG and inhibit its activity. (A) PAGE analysis of DNA assay reaction products in the absence or presence of complexes. RM-1 cell lysates were collected and products treated with UDG in presence of **A8**, UDGI (positive control), or **A2** (negative control), followed by resolution on 20% polyacrylamide gel to separate the cleaved products from the substrate. The separated products were visualized by using ChemiDoc™ MR Imaging System (Bio-Rad), following sliver staining. (B) Stabilization of UDG by **A8***in cellulo*. RM-1 cell lysates were treated with 3 μM of **A8** or DMSO at room temperature for 30 min and then heated at different temperature ranging from 45 °C to 75 °C for 5 min. The supernatants of protein samples were collected and detected by western blotting using UDG antibody. (C) Densitometry analysis of CETSA for the level of remaining soluble protein of UDG at different incubation temperatures for treatment and DMSO-treated control samples. Error bars represent the standard deviations of the results obtained from four independent experiments. (D) Shown are plots of the fluorescence changes of UDG (1000 U mL^−1^) as the temperature was increased in the presence or absence of compound **A8** (10 μM). Error bars represent the standard deviations (SD) of the results from three independent experiments. *P* values were calculated using a two-sided *t*-test. *P < 0.05, **P < 0.01 *vs.* DMSO group.

Non-denaturing polyacrylamide gel electrophoresis (PAGE) was performed to verify the ability of compound **A8** and **S13** to inhibit UDG (Fig. 4A and S5A†). Isolated ON1 and ON2 oligonucleotides migrate at different speeds on the agarose gel (Lanes 1 and 2), and both move faster than the ON1–ON2 duplex (Lane 3). In the presence of UDG from cell lysates, the uracil bases on ON2 will be excised to liberate ON1 and cleaved ON2 (Lane 4), however, this process could be blocked by the presence of both **A8** (Lane 5) or positive control UDGI (Lane 6). **A2**, a negative control compound, was unable to prevent the release of ON1 (Lane 7). These results verify that the decrease in the emission of the G-quadruplex/**1** system by **A8** is likely due to the ability of the compound to inhibit UDG activity rather than *via* other mechanisms, such as emission quenching. In order to distinguish the cleaved DNA band more clearly, we further designed the sequence ON3 containing only a single uracil (5′-CGCACTUA_2_GA_2_T_3_C-3′). PAGE analysis showed that ON1–ON3 duplex (Lane 3) could be cleaved by UDG with or without **A2** (Lane 4 and Lane 7), as revealed by the appearance of the cleaved ON3 band, but that this was blocked by the presence of **A8** (Lane 5) or UDGI (Lane 6) (Fig. S4†). Compared with the band cleaved by UDG with or without A2 (Lane 4 and Lane 7), **S13** could also reduce the cleaved ON3 band (Lane 5), although it slightly less effective than the positive control UDGI (500 U mL^−1^) or **A8** (Fig. S5A†).

In a cellular thermal shift assay (CETSA), obvious shifts in melting curve of UDG in RM-1 advanced prostate cancer cell lysates were observed in the presence of compound **A8** and **S13**, indicating that both of them directly engaged and stabilized UDG (Fig. 4B, C and S5B, C†). These were corroborated using a fluorescence-based protein thermal shift assay (FTSA), which revealed by a marked shift of the melting curve (*ca.* 2.0 °C for **A8** and *ca.* 1.2 °C for **S13**) (Fig. 4D and S5D†).

### Combination studies of UDG inhibitors and 5-FU against prostate cancer

**Fig. 5:**
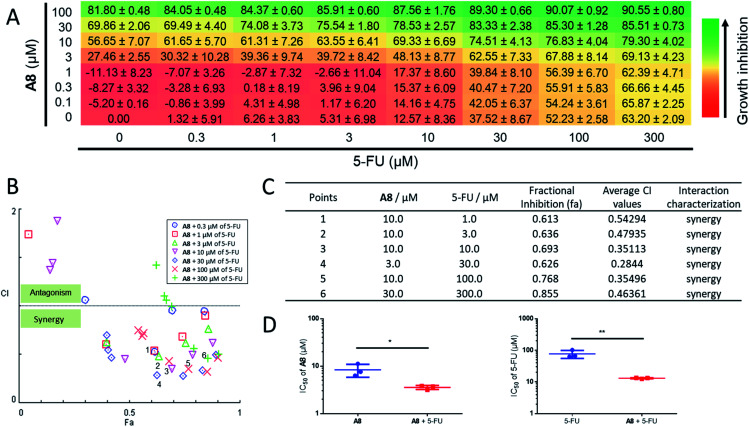
Combination assay for **A8** and 5-FU. RM-1 cells were treated with combinations of **A8** and 5-FU for 48 h and growth inhibition was determined using the MTT assay. (A) Checkerboard data showing viability of RM-1 cells with varying **A8** (0–100 μM) and 5-FU (0–300 μM) concentrations as a percentage of untreated cells. Data are expressed as means ± SD (*n* = 3). (B and C) Combination effect analysis for **A8** and 5-FU. Combination index (CI, measure of drug synergy) was determined using the Chou-Talalay method. CI values of <1 indicate drug synergy. (D) The concentration needed to reach 50% inhibition (IC_50_) of cell proliferation is indicated. The left histograms indicate the IC_50_ of **A8** as a single agent and in combination with 5-FU in the RM-1 cell line. The right histograms indicate the IC_50_ of 5-FU as a single agent and in combination with **A8**. The IC_50_ values for the combination of the two compounds were determined using rays with an effective fraction ∼0.5, corresponding to compounds that are in equipotent proportion (**A8** : 5-FU, 1 : 3) in the mixture. Error bars represent the standard deviations of the results obtained from three independent experiments. *P* values were calculated using a two-sided *t*-test. *P < 0.05, **P < 0.01 UDGI or 5-FU *vs.* UDG + 5-FU group, respectively.

Since UDG depletion is known to sensitize cancer cells to chemotherapy,^93^ the ability of the identified UDG inhibitor **A8** to synergize with the anticancer drug, 5-FU, was next investigated in prostate cancer cells. Using the Chou-Talalay method, significant synergism was shown between **A8** and 5-FU at inhibiting RM-1 cell proliferation (Fig. 5A). In wells with significant synergy for which there were equipotent proportions of **A8** and 5-FU in the mixture, a 2.4-fold lower **A8** concentration (IC_50_ = 3.56 ± 0.35 μM) and 6-fold lower 5-FU concentration (IC_50_ = 13.09 ± 0.46 μM) were required together to achieve 50% growth inhibition, *versus* treatment with each single agent alone (IC_50_ = 8.37 ± 2.55 μM for **A8** and IC_50_ = 77.16 ± 21.84 μM for 5-FU, respectively) (Fig. 5B–D, and Table 1). An apparent synergism was also shown between nifuroxazide and 5-FU with 2.7-fold lower **S13** concentration (IC_50_ = 8.39 ± 0.86 μM) and 3.6-fold lower 5-FU concentration (IC_50_ = 26.33 ± 2.67 μM) to reach 50% growth inhibition, respectively (Fig. S6†). For UDGI, 4.8- and 2.5-fold lower concentrations were needed in combination to reach 50% growth inhibition (IC_50_ = 25.23 ± 2.90 U mL^−1^ for UDGI and IC_50_ = 24.93 ± 3.71 μM for 5-FU) relative to treatment with the single agent for UDGI (IC_50_ = 121.84 ± 31.76 U mL^−1^) and 5-FU (IC_50_ = 61.80 ± 5.04 μM), respectively (Fig. S7†). Collectively, these results indicate that both of **A8** and **S13** could synergize with 5-FU in lowering the proliferation of prostate tumour cells *in cellulo*.

### Interaction between the UDG inhibitor and the binding pocket of UDG

The above studies demonstrated that **A8** and **S13** could inhibit the UDG activity and enhance 5-FU-induced growth inhibition. To further understand the interaction of small molecule inhibitors with UDG, molecular modelling was performed with **A8**, as the more potent of the two compounds. The model of UDG was constructed from the X-ray crystal structure (PDB : 3FCI) of UDG in complex with the reported UDG inhibitor, compound **P** (Fig. S8A†),^16^ using the molecular conversion procedure implemented in the ICM-pro 3.6-1d program (Molsoft). Compound **A8** was predicted to bind snugly in the pocket of UDG (Fig. S8B†), forming hydrogen bonding interactions to Asp145 and Ser247 *via* its sulfonamide and nitro groups respectively. The binding pose of **A8** overlapped significantly with the binding pose of compound **P** with UDG (Fig. S8C†), with the 4-nitrophenyl ring of **A8** occupying roughly the same location as the terminal benzoic acid group of compound **P**. Notably, both compounds form hydrogen bonding interactions with Ser247, although compound **P** also makes additional interactions to Gln144, Phe158, Asn204 and Tyr248 that are not replicated in **A8**. The interaction with Ser247 may be a key determinant for UDG inhibitory activity, as **A2**, which has a similar structure to **A8** but lacks the nitro group that **A8** uses to hydrogen bond to Ser247, showed no activity in the UDG inhibitor assay.

### Inhibition of UDG activity promotes 5-FU-induced DNA damage and cell cycle arrest in advanced prostate cancer cells

UDG expression is coordinated with DNA replication.^94,95^ Two well-recognized molecular indicators of DNA damage, phosphorylation of histone H_2_AX (γH_2_AX), and cleavage of poly(ADP-ribose) polymerase (cleaved-PARP), were then analyzed. Immunoblotting results showed a significant increase (*ca.* 2.5 fold) in the co-treated group compared with only the 5-FU treated group (Fig. S9A†), and an increasing accumulation of Ser139-phosphorylated γ-H_2_AX and cleaved-PARP in the combination group over 0, 4, 8, 12, 16, and 20 h of treatment (Fig. S9B†), suggesting that **A8** could enhance the ability of 5-FU to induce DNA damage. This finding was further confirmed by immunofluorescence analysis, which showed increased expression of γH_2_AX level in the **A8**/5-FU combination group (Fig. S9C†). To visualize DNA damage more clearly, a comet assay was carried out, which showed more long-tail migrated DNA, indicative of DNA damage, in the **A8**/5-FU combination group compared with the single-treatment groups and the control group (Fig. S9D†).

5-FU is well-known to affect the cell cycle.^96,97^ To further verify the synergistic effect of **A8** and 5-FU, and also to evaluate the selectivity of **A8** interacting with UDG in cells, cell cycle analysis combined with siRNA knockdown experiments were performed. As shown Fig. S9E,† the percentages of G0/G1 and S-phase cells were relatively unaffected in the presence of **A8** and 5-FU in UDG knockdown RM-1 cells, while co-treatment of **A8** and 5-FU resulted in a significant decrease of G0/G1 phase cells and an increase in the percentage of S-phase cells. This suggests that the cell cycle modulation by 5-FU is enhanced by **A8** and also requires the presence of UDG in RM-1 cells.

## Discussion

Many biochemical small inhibitor screening assays have been developed based on reactions to produce conventional fluorophores, such as commercial steady-state fluorescent inhibitor screening assay kits.^98^ Moreover, conventional steady-state fluorometric detection is also central to many other screening applications, including, but not limited to, enzyme-linked immunosorbent assay (ELISA), the AlphaScreen assay, flow cytometry and immunohistochemistry (IHC). These inhibitor screening methods provide a very effective technique for high-throughput screening (HTS) of proteins or kinase inhibitors. However, they also share several limitations. Firstly, high background signals are often raised by the simultaneous excitation and emission processes. Secondly, many commercially available dyes suffer from small Stokes shifts, which can result in poor signal-to-noise (S : N) ratios, as well as self-quenching due to overlap between their absorption and emission spectra.^99^ Thirdly, autofluorescent substances of biological matrices (cell or tissue samples) often act as a source of background signal in assays,^100^ because it is very hard to eliminate their presence completely prior to measurement. Finally, the fluorescent nature of certain classes of small-molecule compounds can generate false negatives or positives in a fluorescence-based HTS strategy.^101^ In this study, we employed a privileged library of 400 natural product-like or approved dug compounds covering over 50 types of potential bioactive chemical scaffolds, including saturated and unsaturated heterocycles, β-amino acids, sulfonamides, and carboxylic acids. Significantly, most of these compounds were known to exhibit higher background fluorescence in steady-state mode which makes them unsuitable for most photoluminescence screening methods. Thus, we anticipated that TRES could be an effective strategy to eliminate or even avoid the problem of background fluorescence for the screening of small molecule inhibitors.

Different from standard steady-state fluorometric methods, TRES detection provides an effective strategy to overcome fluorescence interference by recording the luminescence decay profile with high temporal resolution after a pulsed excitation.^102^ TRES relies on the use of specific luminescent probes, such as iridium complexes, that have long-lived emission which allows detection of the emitted light to take place long after excitation has occurred. Specifically, the long luminescence lifetime (typically microseconds) of iridium probes are usually several orders of magnitude greater than nonspecific background fluorescence (typically nanoseconds), which enables the emission to be read at a time well after any background fluorescence has decayed. Iridium probes also have large Stokes shifts, which can also help to increase the S : N ratio.^103^ Finally, the high luminescence intensity and stable emission signal of iridium probes also significantly improves assay sensitivity, robustness and dynamic range.

UDG is emerging as a very interesting pharmacologic target for therapeutic intervention due to its important function in regulating various physiological activities, including DNA replication within viruses, and the generation of DNA strand breaks during chemotherapy.^13^ Many cancers have developed resistance to 5-FU, due to the ability of UDG to continuously repair 5-FU induced DNA damage.^104,105^ Therefore, the combination of 5-FU with a UDG inhibitor could be a potential strategy to overcome 5-FU resistance and generate effective anticancer effect, including against prostate cancer. Several potent small molecule UDG inhibitors with IC_50_ values between 1.1 to 315 μM have been identified by screening linked libraries using 6-formyl uracil as the substrate fragment.^13,16,17^ However, none of these have undergone further in-depth disease application research, such as the treatment of drug-resistant cancers, including in combination therapy with clinical drugs. In addition, besides the general strategy of substrate fragment tethering, effective methods for the discovery of novel UDG inhibitors are still lacking. In this work, we have demonstrated for the first time the use of TRES to overcome the background fluorescence of samples in order to identify UDG inhibitors from a privileged library of natural product/natural product-like compounds. The method exploits the long phosphorescence lifetime (931 ns) of iridium probe **1**, which is much longer than the fluorescence lifetimes of library. Using a delay time of 500 ns in TRES mode, the emission of the complex **1** could be clearly distinguished and the problem of false negatives in steady-state emission mode could be avoided.^38^ In particular, we note that the promising UDG inhibitors **A8** and **S13** identified in this work would have been recorded as a non-active under steady-state mode, whereas both of them showed clear UDG inhibition *via* TRES. One drawback of this strategy is that it is relatively time-consuming compared with traditional steady-state fluorescent screening method. Moreover, while it can overcome interference from short-lived background fluorescence, it is still susceptible to interference from inhibitors with longer fluorescence lifetimes.

The indole derivative **A8** showed 81% inhibition of UDG activity at 10 μM, compared to 65% for UDGI and 79% for **S13**, and displayed an IC_50_ value of 1.9 μM against UDG activity in a dose–response experiment. More importantly, we demonstrated that inhibition of UDG activity by **A8** and **S13** significantly promotes the anti-tumor effect of 5-FU in advanced prostate cancer cells. Non-denaturating PAGE analysis showed that both of them compromised the ability to cleave ON2 and liberate ON1, consistent with the proposed mechanism of the assay. Molecular docking analysis further elaborated that the high affinity of lead compound **A8** binding to binding pocket of UDG by forming two H-bondings and hydrophobic effect with the binding pocket of UDG.

Biological experiments showed the potential of indole derivative **A8** and furan derivative **S13** to synergize with 5-FU for prostate cancer therapy. In the checkerboard assay, **A8** and **S13** could synergize with 5-FU in lowering the proliferation of prostate tumour cells *in cellulo*. Moreover, comparison of IC_50_ fold-changes suggested that **A8** might be better than UDGI at synergizing with 5-FU. However, it should be noted that the *in vitro* potency of these compounds against UDG kinase can be different from their *in cellulo* activities, due to variations in cell absorption, metabolism, or other factors. The combination of **A8** and 5-FU increased DNA damage markers, γ-H_2_AX and cleaved-PARP, in RM-1 cells, suggesting that **A8** could enhance the ability of 5-FU to induce DNA damage. This finding was further confirmed by immunofluorescence analysis and a comet assay. Meanwhile, the cell cycle data showed that **A8** could enhance the cell cycle regulation of 5-FU by selective inhibition of UDG activity in RM-1 cells.

In conclusion, we have successfully developed a TRES-based UDG activity detection method that can overcome the background fluorescence of samples. This work led to the identification of the indole derivative **A8** and the FDA/EMA-approved drug **S13**, as only the second and third classes overall of UDG small molecule inhibitors reported to date. Moreover, **A8** showed comparable potency to the report UDG small molecule inhibitor, and also showed promising synergism with 5-FU at inducing DNA damage and impairing the proliferation of prostate cancer cells. Notably, drug **S13** has anti-cancer properties that have been elucidated in previous studies,^106–108^ and the discovery that **S13** targets UDG target will facilitate drug repurposing against resistance of antineoplastic drugs. We envision that this rapid screening method could be easily adapted for the screening of other BER enzyme inhibitors for potential therapeutic applications, including previously neglected scaffolds classes that could not be screened because of their high background fluorescence leading to false negatives.

## Experimental

### Cells and reagents

Dulbecco's modified Eagle's medium (DMEM) and Fetal bovine serum (FBS) were obtained from Gibco BRL (Gaithersburg, MD, USA). UDG and UDG inhibitor (UDGI) were obtained from New England Biolabs Inc. All oligonucleotides were synthesized by IGE Bio Inc (Guangzhou, China), and the sequences were as follows: ON1: 5′-GGGTAGGGAAATTCTTAAGTGCGGGTTGGG-3′; ON2: 5′-CGCACUUAAGAAUUT C-3′. All the antibodies were obtained from Cell Signaling Technology. Luciferase assay reagent and passive lysis buffer were purchased from Promega Corporation (Madison, WI, USA). All the natural compounds were provided by Prof. Philip Wai Hong Chan (Monash University) in dimethyl sulfoxide (DMSO) at a stock concentration of 10 mM. Complex **1** was synthesized according to a literature method.^109^

### UDG inhibitor screening

The solution containing ON1 (100 μM) and ON2 (100 μM) sequences in a hybridization buffer (50 mM Tris, 150 mM NaCl, pH 7.0), was heated to 95 °C for 10 min and then cooled at 0.1 °C s^−1^ to room temperature to allow the formation of the duplex substrate (ON1–ON2). The annealed product was stored at −20 °C before use. The duplex substrate was incubated with the indicated concentrations of UDG and 10 μM natural product-like compounds. The mixture was heated to 37 °C for 30 min to allow the base cleavage reaction to take place. The mixture was cooled down and was subsequently diluted using Tris buffer (50 mM Tris, 20 mM KCl, 150 mM NH_4_OAc, pH 7.0) to a final volume of 500 μL. Complex **1** was added to a final concentration of 0.5 μM. Steady-state photoluminescence spectra were obtained in a HORIBA Fluorolog-3 spectrophotometer, with excitation at 355 nm. Time-resolved studies were performed using a time-correlated single photon counting (TCSPC) technique on this Fluorolog-3 spectrophotometer using a delay time of 500 ns. The use of a 500 ns delay time eliminates the short-lived fluorescence of the screened compounds, while the long-lived phosphorescence of complex **1** remains to be detected.^110^ The luminescence emission intensity at 450–700 nm was then monitored after excitation of the sample at 355 nm.

### DNA polyacrylamide gel electrophoresis

This experiment was carried as previously described with minor modification.^111^ The UDG-treated product was obtained according to the procedure described above. The products were resolved on 20% polyacrylamide gel to separate the cleaved products from the substrate using 1× TBE as running buffer at a constant voltage of 100 V for 120 min. The separated products were visualized by using the ChemiDoc™ MR Imaging System (Bio-Rad), following silver staining according to protocol of Fast Sliver Stain kit for Nucleic Acids (Real-Times, China).

### Immunofluorescence assay

The cell damage and prolonged mitosis were analyzed according to previous methods with minor modifications.^112^ RM-1 cells, an advanced prostate cancer cell line, were seeded in 35 mL plates at a density of (6 × 10^4^) cells per mL overnight and treated with DMSO, **A8** (3.0 μM), 5-FU (10.0 μM) and the combination treatment for 12 h. As for γH_2_AX, after fixation in 4% paraformaldehyde and permeabilization in 0.2% Triton X-100 in PBS for 30 minutes. Cells were incubated with fluorescein isothiocyanate (FITC)-conjugated mouse anti-γH_2_AX(Ser139) monoclonal antibody (1 : 500) (BioLegend) overnight. Cell nuclei were counterstained with 4,6-diaidino-2-phenylindole (DAPI), and then stained with DAPI for 15 min. The photos and density values of fluorescence were acquired on a Leica confocal microscope.

### Combination assay

RM-1 (5000 cells per well) were seeded in 96-well plates with regular media and allowed to adhere for 24 h. The following day, the media was replaced with serum free media and cells were treated as indicated for an additional 48 h. Cell proliferation was measured using the MTT method. 20 μL 5× MTT was added in each well, and the wells were incubated at 37 °C for 4 h. The absorbance at 570 nm was measured with a plate reader. Each experiment was performed in triplicate, and the experiments are shown with standard deviation from the experiment as error bars. The error bars in reported IC_50_ values represent the standard deviation from three different experiments. Combination index (CI) was determined using the Chou-Talalay method.^113^

## Author contributions

G. L., T. S. K., J. T. Z., and S. C. N. carried out the *in vitro* and *in cellulo* experiments. G. L., T. S. K., H. L., and C. W. wrote the manuscript. S. A. H., Y. Z. and J. J. carried out synthesis complexes. P. W. H. C., D. L. M. and C. H. L. designed the experiments and analyzed the results.

## Conflicts of interest

There are no conflicts of interest to declare.

## Supplementary Material

SC-011-C9SC05623H-s001

SC-011-C9SC05623H-s002
