# Supplementary material for: A robust photoluminescence screening assay identifies uracil-DNA glycosylase inhibitors against prostate cancer
Source: Chem Sci. 2020 Jan 10;11(7):1750–60. doi: 10.1039/c9sc05623h (PMC8148385; doi:10.1039/c9sc05623h)
Supplement: SC-011-C9SC05623H-s002 [file SC-011-C9SC05623H-s002.pdf]

Electronic Supplementary Material (ESI) for Chemical Science.
This journal is © The Royal Society of Chemistry 2020

Supporting Information

A robust photoluminescence screening assay identifies uracil-DNA glycosylase inhibitors against prostate cancer†

Guodong Li,‡[a] Stuart Adam Henry,‡[b] Hao Liu,‡[c] Tian-Shu Kang,‡[a] Sang-Cuo Nao,‡[a] Yichao Zhao,[d] Chun Wu,[c] Jianwen Jin,[d] Jia-Tong Zhang,[a] Chung-Hang Leung,*[a] Philip Wai Hong Chan*[b,d] and Dik-Lung Ma*[c]

[a] State Key Laboratory of Quality Research in Chinese Medicine, Institute of Chinese Medical Sciences, University of Macau, Macao

[b] Department of Chemistry, University of Warwick, Coventry, UK

[c] Department of Chemistry, Hong Kong Baptist University, Hong Kong

[d] School of Chemistry, Monash University, Clayton, Australia

‡ These authors contributed equally to this work.

* To whom correspondence should be addressed. Tel: (+853) 8822-4688; Fax: (+853) 2884-1358; Email: duncanleung@um.edu.mo

Correspondence may also be addressed to Dik-Lung Ma and Philip Wai Hong Chan. Tel: (+852) 3411-7075; Email: edmondma@hkbu.edu.hk; Tel: (+61) 3 9905 1337; Email: phil.chan@monash.edu

Experimental section

General experimental procedure for the preparation of the compounds

Mass spectrometry was performed at the Mass Spectroscopy Unit at the Department of Chemistry, Hong Kong Baptist University, Hong Kong (China). Deuterated solvents for NMR purposes were obtained from Armar and used as received. Circular dichroism (CD) spectra were collected on an OLIS 1000 CD Spectrometer (OLIS, Jefferson, GA). Drugs were purchased from MedChem Express (Monmouth Junction, NJ).

Synthesis

The following complexes were prepared according to (modified) literature methods:

A1–**A9**: Reported[1]

B1–**B4** and **C1**–**C5**: Reported[2]

D1–**D16**: Reported[3]

G1–**G8**: Reported[4]

H1–**H9**: Reported[5]

DNA sequences used in this project:

Name	Sequences
ssDNA	5′- $G_3T_2ACTACGA_2CTG_2$-3′
ds26	5'-$CA_2TCG_2ATCGA_2T_2CGATC_2GAT_2G$-3'
ds17	5′-$C_2AGT_2CGTAGTA_2C_3$-3′<br>5′-$G_3T_2ACTACGA_2CTG_2$-3′
c-kit87	5′- $AG_3AG_3CGCTG_3AG_2AG_3$-3′

Luminescence response of complex 1 towards different forms of DNA sequences

The sequences ssDNA, ds26, ds17, c-kit87 and ON1 were annealed in Tris-HCl buffer (20 mM Tris, 100 mM KCl, pH 7.4) and were stored at -20 °C before use. Complex **1** (1 μM) was added to 5 μM of ds17, ON1-ON2 or ON1 in Tris-HCl buffer (20 mM Tris-HCl, pH 7.4), then their emission intensity were tested. Emission spectra were recorded in the 500−700 nm range using an excitation wavelength of 355 nm at room temperature.

Cellular thermal shift assay

Cellular thermal shift assay was performed to monitor the target engagement of **A8** in RM-1 cell lysates. Briefly, cell lysates from 2 × 10^6 RM-1 cells were collected, diluted in PBS and separated in the same aliquots. Each aliquot was treated with 3 μM **A8** or DMSO. 30 min after incubation at room temperature, the compound-treated lysates were divided into 50 μL in each of PCR tubes and heated individually at different temperatures (Veriti thermal cycler, Applied Biosystems/Life Technologies). The heated lysates were centrifuged and the supernatants were analyzed by SDS-PAGE followed by immunoblotting analysis by probing with the indicated antibody.

Comet assay

The comet assay was performed as in previous report with minor revisions.[6] Briefly, microscopic slides were coated with 1% normal agarose (GE Healthcare) followed by adding 1% low melting point (LMP) agarose onto each slide and then covering slides with coverslips. After cooling, the coverslips were removed and the slides were lowered into freshly made pre-chilled lysis buffer for 1 h. Then set the power voltage to 25 V and adjust the current to 300 mA for 20 min to perform the electrophoresis procedures. Cells were stained with PI. Individual cells were viewed using Olympus IX73 fluorescence microscope.

Western blotting

Treated cells were washed with PBS twice and then harvested using ice-cold RIPA lysis buffer containing protease inhibitor cocktail (Gibco). The lysates were centrifuged at 12, 500 g for 20 min at 4 °C and the supernatant fractions were collected. Protein concentrations were measured with BCA Protein Assays Kits (Gibco). After denaturation at 95 °C for 10 min, equivalent aliquots of protein samples (30 μg) were loaded and electrophoresed on SDS-PAGE gels and then transferred to PVDF membrane (Thermo Scientific). The membranes were firstly blocked with 5% nonfat dry milk for 2 h at room temperature and then incubated for 4 h at room temperature. The bands were visualized with the ChemiDocTM MR Imaging System (Bio-Rad).

UDG knockdown assay

RM-1 cells were seeded in 6-well plates at 80% confluence in DMEM medium for 24 h. Lipo3000 reagent and UDG siRNA were gently mixed and the wells were incubated for 20 min at room

temperature. The growth medium was removed from cells, and replaced with 0.5 mL of fresh medium. Then the mixture 500 μL were added to each well. Cells were incubated at 37 °C in a CO_2 incubator for 48 h post-transfection before the further research. The siRNA sequences refer to previous report.[7]

Cell cycle detection

Propidium iodide (PI) staining was conducted for cell cycle detection. Following 3.0 μM **A8**, 10 μM and combination group treatment for 6 h, harvested cells were washed in ice-cold PBS, fixed in 70% ethanol and stored at -20 °C overnight. Vehicle-treated cells were used as the control group. The RM-1 cell pellets were then incubated with RNase A (100 μg/mL) (Sigma), PI (50 μg/mL) (Sigma) and 0.05% Triton X-100. Cellular DNA content was detected on a FACS Calibur (BD Bioscience) flow cytometer.

Molecular docking

The molecular docking was performed as per previous reports.[8] The initial model of UDG was constructed from the X-ray crystal structure of UDG in complex with the reported UDG inhibitor, compound **P** (PDB: 3FCI),[9] using the molecular conversion procedure implemented in the ICM-pro 3.6-1d program (Molsoft).

Fluorescence-based thermal shift assay

The fluorescence-based thermal shift assay was performed using a Protein Thermal Shift Dye Kit (Life Technologies, Cat. No. 4461146) and an Agilent Mx3005p qPCR system (Agilent Technologies, USA) according to the manufacturer's instructions. Briefly, 17.5 μl of UDG solution (optimized final concentration, 1000 U/mL) and 2.5 μl of Protein Thermal Shift Dye 8X were first was loaded onto a 96-well PCR plate. After shaking and centrifugation, the mixture was then subjected to thermal scanning (25 to 95 °C at 1 °C/min), and fluorescence intensity was measured after every 1 min.

Table S1 CI values for non-constant combination analysis (**A8** and 5-FU) by using the Chou-Talalay method.

**A8** / μM	5-FU / μM	Fractional Inhibition (fa)	Average CI values	Interaction characterization	Points
3.0	0.3	0.303	1.06462	antagonism	
10.0	0.3	0.617	0.52645	synergy	
30.0	0.3	0.695	0.9528	synergy	
100.0	0.3	0.84	0.94457	synergy	
0.1	1.0	0.043	1.73995	antagonism	
0.3	1.0	0.0020	327.678	antagonism	
3.0	1.0	0.394	0.60563	synergy	
10.0	1.0	0.613	0.54294	synergy	1
30.0	1.0	0.741	0.68614	synergy	
100.0	1.0	0.844	0.90481	synergy	
0.1	3.0	0.012	21.6951	antagonism	
0.3	3.0	0.04	5.81941	antagonism	

3.0	3.0	0.397	0.62891	synergy	
10.0	3.0	0.636	0.47935	synergy	2
30.0	3.0	0.755	0.61976	synergy	
100.0	3.0	0.859	0.76296	synergy	
0.1	10.0	0.142	1.37125	antagonism	
0.3	10.0	0.154	1.44573	antagonism	
1.0	10.0	0.174	1.87772	antagonism	
3.0	10.0	0.481	0.45651	synergy	
10.0	10.0	0.693	0.35113	synergy	3
30.0	10.0	0.785	0.49487	synergy	
100.0	10.0	0.876	0.61939	synergy	
0.1	30.0	0.42	0.46467	synergy	
0.3	30.0	0.405	0.54432	synergy	
1.0	30.0	0.398	0.70144	synergy	
3.0	30.0	0.626	0.2844	synergy	4
10.0	30.0	0.745	0.28231	synergy	
30.0	30.0	0.833	0.33363	synergy	
100.0	30.0	0.893	0.49537	synergy	
0.1	100.0	0.542	0.74867	synergy	
0.3	100.0	0.559	0.69358	synergy	
1.0	100.0	0.564	0.72429	synergy	
3.0	100.0	0.679	0.42918	synergy	
10.0	100.0	0.768	0.35496	synergy	5
30.0	100.0	0.853	0.32146	synergy	
100.0	100.0	0.901	0.465	synergy	
0.1	300.0	0.659	1.10686	antagonism	
0.3	300.0	0.667	1.05935	antagonism	
1.0	300.0	0.624	1.42096	antagonism	
3.0	300.0	0.691	0.9932	synergy	
10.0	300.0	0.793	0.56439	synergy	
30.0	300.0	0.855	0.46361	synergy	6
100.0	300.0	0.906	0.50202	synergy	

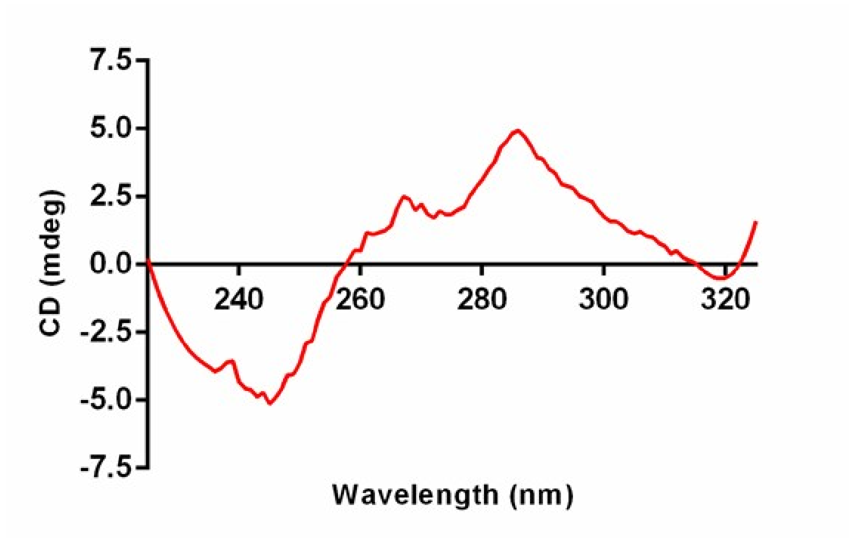

Fig. S1 CD spectra of ON1 G-quadruplex (5 μM) in Tris-HCl buffer (20 mM Tris-HCl, 75 mM KCl, pH 7.4) at room temperature.

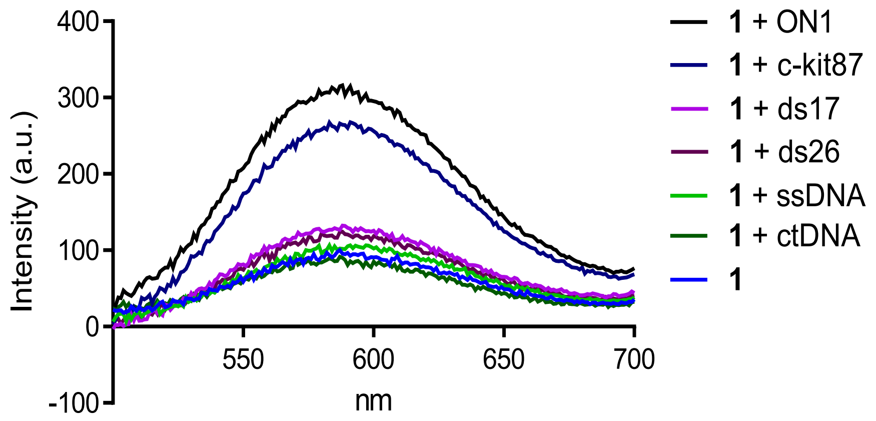

Fig. S2 Emission spectrum of complex **1** (1 μM) in the presence of 5 μM of ctDNA, ssDNA, ds26 (duplex DNA), ds17 (duplex DNA), c-kit87 (G-quadruplex), or ON1.

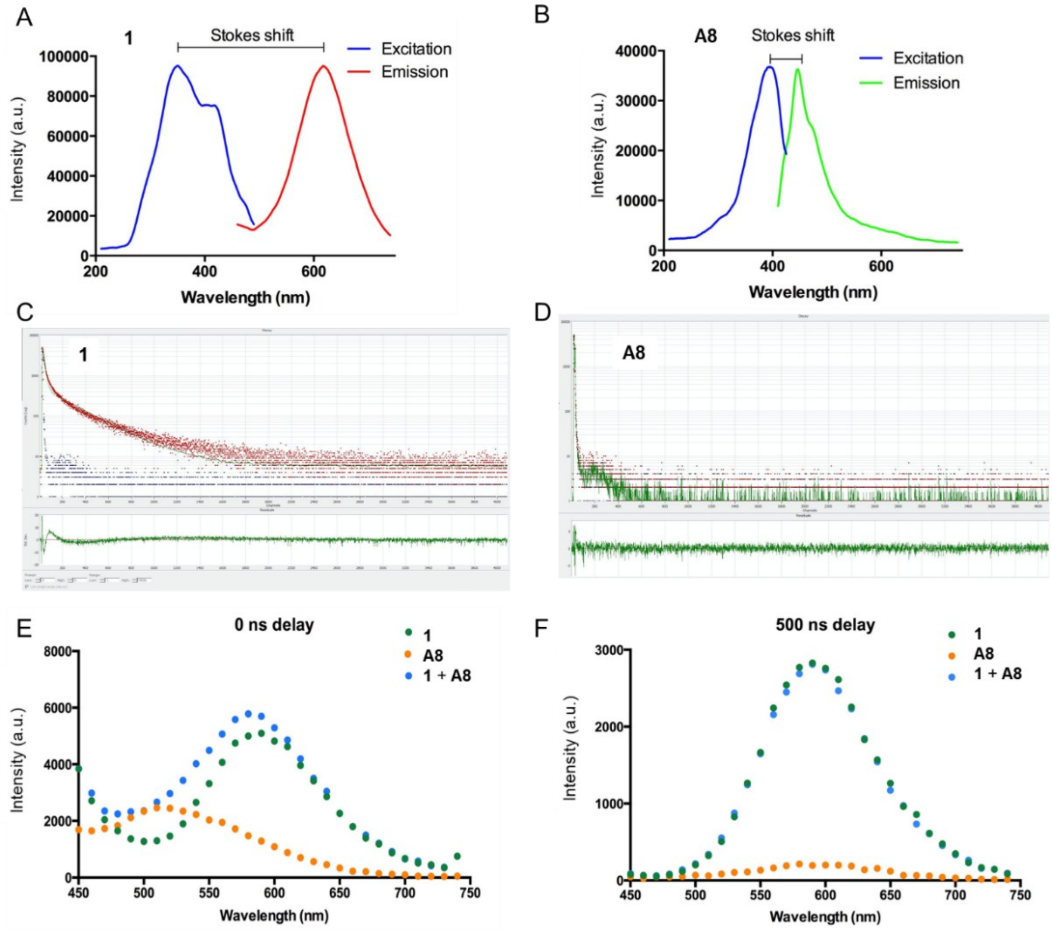

Fig. S3 Photophysical characterization of **1** and **A8**. (A) Excitation and emission spectra of iridium complex **1**. (B) Excitation and emission spectra of **A8**. The decay curve of iridium complex **1** (C) and **A8** (D). Time-resolved spectra of complex **1** and/or **A8** with time gate (E) 0 ns delay or (F) 500 ns delay with excitation at 355 nm.

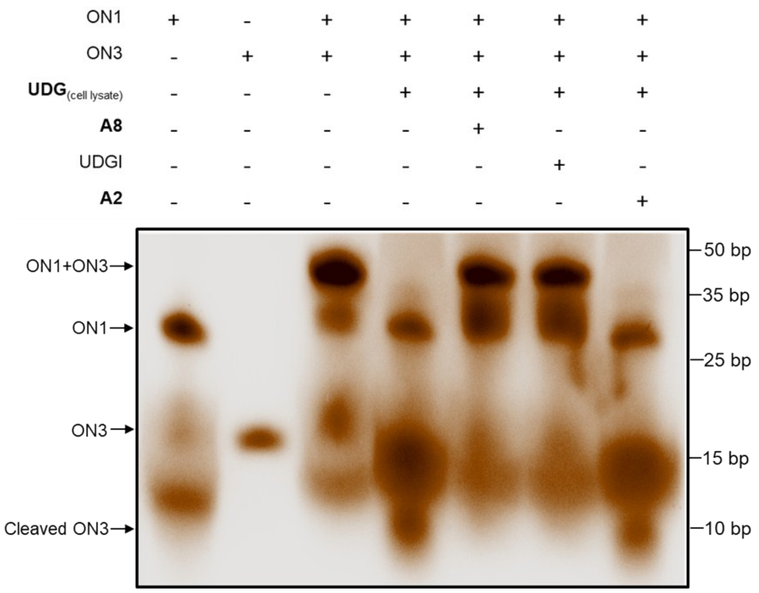

Fig. S4 PAGE analysis of DNA assay reaction products. G-quadruplex-forming motif (ON1, 5'-$G_3TAG_3A_3T_2CT_2A_2GTGCG_3T_2G_3$-3') is hybridized initially with a partly complementary, uracil-containing DNA sequence (ON3: 5'-$CGCACTUA_2GA_2T_3C$-3') to form a double-stranded DNA substrate. RM-1 cell lysates were collected and products treated with UDG in presence of **A8**, UDGI (positive control), or **A2** (negative control), followed by resolution on 20% polyacrylamide gel to separate the cleaved products from the substrate. The separated products were visualized by using ChemiDocTM MR Imaging System (Bio-Rad), following sliver staining.

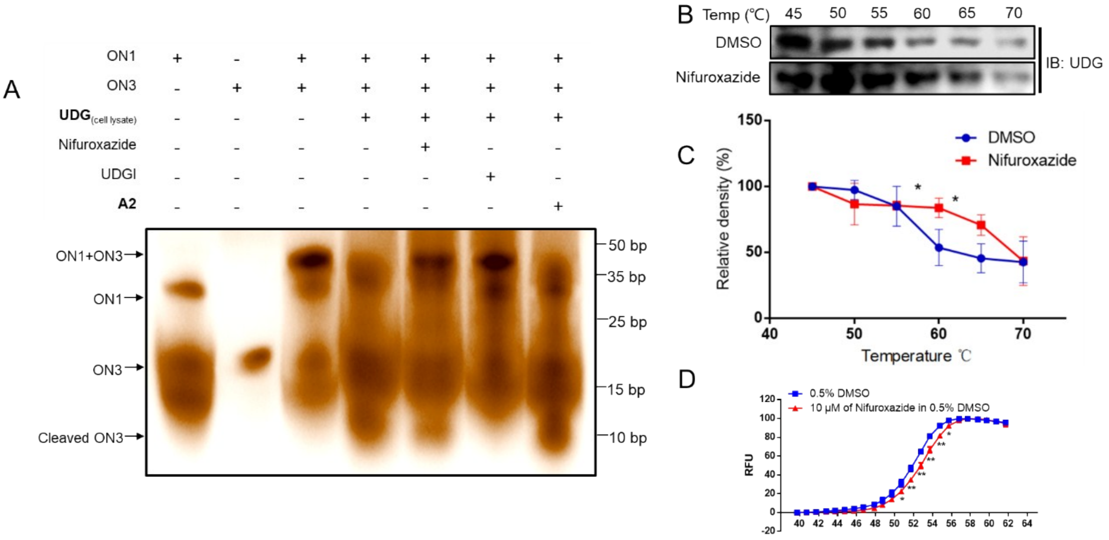

Fig. S5 Nifuroxazide (**S13**) could engage UDG and inhibit its activity. (**A**) PAGE analysis of DNA assay reaction products in the absence or presence of complexes. RM-1 cell lysates were collected and products treated with UDG in presence of nifuroxazide, UDGI (positive control), or **A2** (negative control), followed by resolution on 20% polyacrylamide gel to separate the cleaved products from the substrate. The separated products were visualized by using ChemiDocTM MR Imaging System (Bio-Rad), following sliver staining. (**B**) Stabilization of UDG by nifuroxazide *in cellulo*. RM-1 cell lysates were treated with 10 μM of nifuroxazide or DMSO at room temperature for 30 min and then heated at different temperature ranging from 45 °C to 75 °C for 5 min. The supernatants of protein samples were collected and detected by Western blotting using UDG antibody. (**C**) Densitometry analysis of CETSA for the level of remaining soluble protein of UDG at different incubation temperatures for treatment and DMSO-treated control samples. Error bars represent the standard deviations of the results obtained from four independent experiments. (**D**) Shown are plots of the fluorescence changes of UDG (1000 U/mL) as the temperature was increased in the presence or absence of nifuroxazide (10 μM) by using FTSA. Error bars represent the standard deviations (SD) of the results from three independent experiments. *P* values were calculated using a two-sided t-test. $^{*}P < 0.05$, $^{**}P < 0.01$ vs. DMSO group.

A

Nifuroxazide (μM) \ 5-FU (μM)	0	0.3	1	3	10	30	100	300
100	90.06 ± 0.17	89.15 ± 0.49	88.99 ± 0.19	89.26 ± 0.15	89.50 ± 0.25	89.53 ± 0.22	89.70 ± 0.21	89.98 ± 0.20
30	58.30 ± 3.82	56.45 ± 5.47	56.19 ± 4.29	55.84 ± 6.19	56.24 ± 5.78	59.90 ± 2.91	70.31 ± 3.87	77.65 ± 3.98
10	27.92 ± 9.02	26.16 ± 6.58	29.76 ± 8.88	32.01 ± 11.37	43.52 ± 1.25	52.57 ± 1.47	68.04 ± 3.96	71.79 ± 5.78
3	4.53 ± 0.80	3.83 ± 0.98	12.78 ± 3.20	19.78 ± 7.25	33.49 ± 2.60	44.31 ± 1.60	59.83 ± 2.09	65.67 ± 4.78
1	-4.85 ± 2.59	-0.32 ± 4.14	11.58 ± 1.51	15.89 ± 0.62	24.35 ± 10.58	43.43 ± 2.22	56.76 ± 5.98	63.46 ± 2.31
0.3	3.19 ± 11.77	-1.27 ± 0.75	7.78 ± 4.32	9.37 ± 8.48	19.03 ± 12.54	40.79 ± 1.52	53.28 ± 7.35	65.32 ± 6.00
0.1	-2.19 ± 2.70	-0.75 ± 3.17	6.47 ± 1.27	5.72 ± 4.34	13.42 ± 7.71	35.08 ± 8.33	50.12 ± 5.64	60.45 ± 0.97
0	0.00	2.37 ± 2.42	2.29 ± 3.67	6.39 ± 2.52	17.22 ± 5.05	35.55 ± 0.91	51.50 ± 7.25	60.01 ± 2.17

Growth inhibition

B

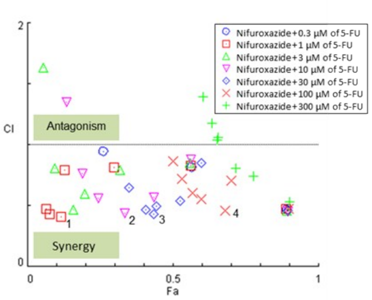

C

Points	Nifuroxazide / μM	5-FU / μM	Fractional Inhibition (fa)	Average CI values	Interaction characterization
1	1.0	1.0	0.116	0.41027	synergy
2	3.0	10.0	0.335	0.43670	synergy
3	1.0	30.0	0.407	0.42825	synergy
4	10.0	100.0	0.680	0.45664	synergy

D

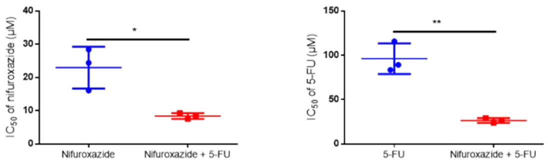

Fig. S6 Combination assay for nifuroxazide (**S13**) and 5-FU. RM-1 cells were treated with combinations of nifuroxazide and 5-FU for 48 h and growth inhibition was determined using the MTT assay. (**A**) Checkerboard data showing viability of RM-1 cells with varying nifuroxazide (0-100 μM) and 5-FU (0-300 μM) concentrations as a percentage of untreated cells. Data are expressed as means ± SD (n = 3). (**B** and **C**) Combination effect analysis for nifuroxazide and 5-FU. Combination index (CI, measure of drug synergy) was determined using the Chou-Talalay method. CI values of <1 indicate drug synergy. (**D**) The concentration needed to reach 50% inhibition (IC_{50}) of cell proliferation is indicated. The left histogram indicates the IC_{50} of nifuroxazide as a single agent and in combination with 5-FU in the RM-1 cell line. The right histogram indicates the IC_{50} of 5-FU as a single agent and in combination with nifuroxazide. The IC_{50} values for the combination of the two compounds were determined using rays with an effective fraction~0.5, corresponding to compounds that are in equipotent proportion (**S13**:5-FU, 1:3) in the mixture. Error bars represent the standard deviations of the results obtained from three independent experiments. *P* values were calculated using a two-sided t-test. *P < 0.05, **P < 0.01 nifuroxazide or 5-FU vs. nifuroxazide + 5-FU group, respectively.

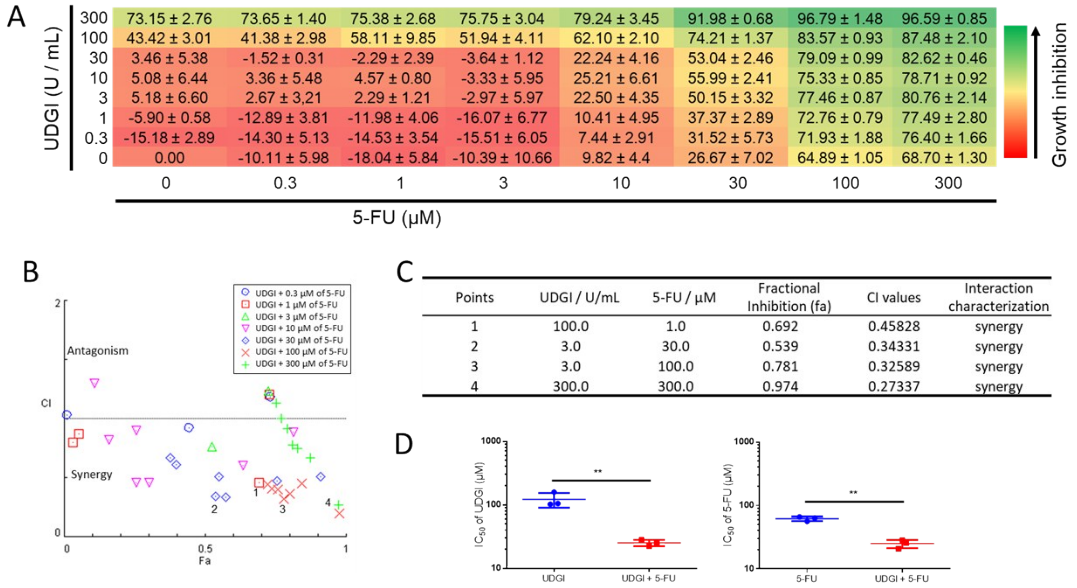

UDGI (U / mL) \ 5-FU (μM)	0	0.3	1	3	10	30	100	300
300	73.15 ± 2.76	73.65 ± 1.40	75.38 ± 2.68	75.75 ± 3.04	79.24 ± 3.45	91.98 ± 0.68	96.79 ± 1.48	96.59 ± 0.85
100	43.42 ± 3.01	41.38 ± 2.98	58.11 ± 9.85	51.94 ± 4.11	62.10 ± 2.10	74.21 ± 1.37	83.57 ± 0.93	87.48 ± 2.10
30	3.46 ± 5.38	-1.52 ± 0.31	-2.29 ± 2.39	-3.64 ± 1.12	22.24 ± 4.16	53.04 ± 2.46	79.09 ± 0.99	82.62 ± 0.46
10	5.08 ± 6.44	3.36 ± 5.48	4.57 ± 0.80	-3.33 ± 5.95	25.21 ± 6.61	55.99 ± 2.41	75.33 ± 0.85	78.71 ± 0.92
3	5.18 ± 6.60	2.67 ± 3,21	2.29 ± 1.21	-2.97 ± 5.97	22.50 ± 4.35	50.15 ± 3.32	77.46 ± 0.87	80.76 ± 2.14
1	-5.90 ± 0.58	-12.89 ± 3.81	-11.98 ± 4.06	-16.07 ± 6.77	10.41 ± 4.95	37.37 ± 2.89	72.76 ± 0.79	77.49 ± 2.80
0.3	-15.18 ± 2.89	-14.30 ± 5.13	-14.53 ± 3.54	-15.51 ± 6.05	7.44 ± 2.91	31.52 ± 5.73	71.93 ± 1.88	76.40 ± 1.66
0	0.00	-10.11 ± 5.98	-18.04 ± 5.84	-10.39 ± 10.66	9.82 ± 4.4	26.67 ± 7.02	64.89 ± 1.05	68.70 ± 1.30

Points	UDGI / U/mL	5-FU / μM	Fractional Inhibition (fa)	CI values	Interaction characterization
1	100.0	1.0	0.692	0.45828	synergy
2	3.0	30.0	0.539	0.34331	synergy
3	3.0	100.0	0.781	0.32589	synergy
4	300.0	300.0	0.974	0.27337	synergy

Fig. S7 Combination assay for UDGI and 5-FU. RM-1 cells were treated with combinations of UDGI and 5-FU for 48 h and growth inhibition was determined using the MTT assay. (**A**) Checkerboard data showing viability of RM-1 cells with varying UDGI (0-300 U/mL) and 5-FU (0-300 μM) concentrations as a percentage of untreated cells. Data are expressed as means ± SD (n = 3). (**B** and **C**) Combination effect analysis for UDGI and 5-FU. Combination index (CI, measure of drug synergy) was determined using the Chou-Talalay method. CI values of <1 indicate drug synergy. (**D**) The concentration needed to reach 50% inhibition (IC_{50}) of cell proliferation is indicated. The left histogram indicates the IC_{50} of UDGI as a single agent and in combination with 5-FU in the RM-1 cell line. The right histogram indicates the IC_{50} of 5-FU as a single agent and in combination with UDGI. The IC_{50} values for the combination of the two compounds were determined using rays with an effective fraction~0.5, corresponding to compounds that are in equipotent proportion (UDGI: 5-FU, 1 U/mL: 1 μM) in the mixture. Error bars represent the standard deviations of the results obtained from three independent experiments. *P* values were calculated using a two-sided t-test. **P* < 0.05, ***P* < 0.01 UDGI or 5-FU vs. UDGI + 5-FU group, respectively.

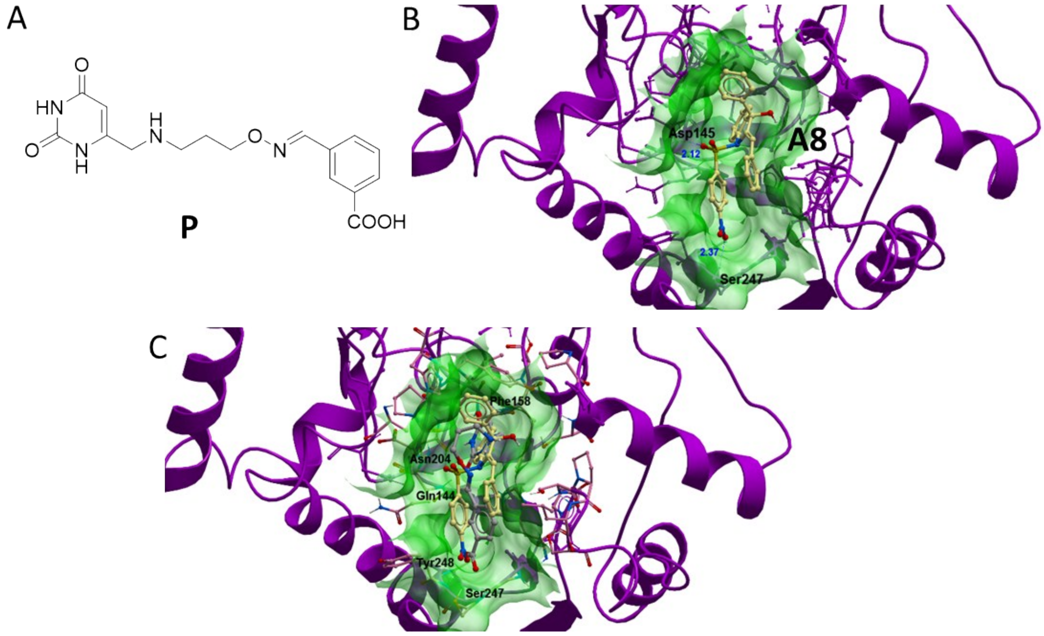

Fig. S8 Top view of **1a** bound to UDG generated by molecular docking. (A) Molecular structure of reported UDG inhibitor compound **P**. (B) UDG (PDB: 3FCI) is depicted as a space-filling representation showing carbon (yellow), oxygen (red), nitrogen (blue) atoms. The binding pocket of the UDG is represented as a translucent green surface. Minimized pose of **1a** by in silico docking. H-bonds are indicated as blue lines and the metal center as a green sphere. (C) The comparison of the binding mode between **A8** and **P**.

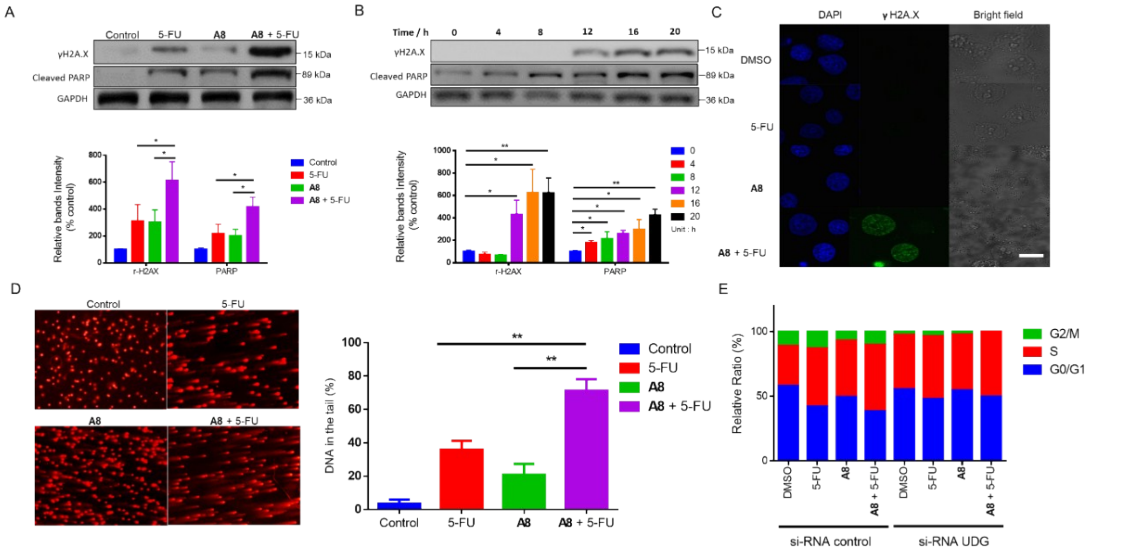

Fig. S9 **A8** and 5-FU treatment causes DNA damage in RM-1 advanced prostate cancer cells. (A) RM-1 cells were treated with DMSO, **A8** (3 μM), 5-FU (10 μM), and the combination group for 12 hour, and analyzed by Western blotting with densitometry quantification. (B) Effect of combination **A8** (3 μM) and 5-FU (10 μM) on the levels of γ-H2A.X and cleaved-PARP proteins in RM-1 cells. RM-1 cells were treated with 3 μM of **A8** and 5-FU for 0, 4, 8, 12, 16, 20 hours, and analyzed by Western blotting with densitometry quantification. (C) RM-1 cells were treated with DMSO, **A8** (3 μM), 5-FU (10 μM), and the combination group for 12 h and visualized using a confocal laser scanning microscope. Scale bar = 20 μM. (D) Evaluation of DNA damage by the comet assay. Images of different treated and untreated samples showing comet formation. The graph represents the comet lengths of different treated and untreated samples randomly selected from their respective group of comet data obtained by analyzing the images. Data presented here are as a percentage of control. (E) Cell cycle analysis of compound **A8** and 5-FU combination on RM-1 cells with or without UDG siRNA treatment. RM-1 cells were first incubated with UDG siRNA or control siRNA for 24h, and then treated with DMSO, **A8** (3 μM), 5-FU (10 μM), and the combination group for 12 hours, respectively. The percentage distribution of cells in the G1, S and G2/M phases are then calculated. *P* values were calculated using a two-sided t-test. $^{*}P < 0.05$, $^{**}P < 0.01$ vs. vehicles, respectively.

References

1. D. Susanti, F. Koh, J. A. Kusuma, P. Kothandaraman and P. W. H. Chan, *The Journal of organic chemistry*, 2012, **77**, 7166-7175.
2. W. Rao, M. J. Koh, P. Kothandaraman and P. W. H. Chan, *Journal of the American Chemical Society*, 2012, **134**, 10811-10814.
3. T. M. U. Ton, C. Tejo, D. L. Y. Tiong and P. W. H. Chan, *Journal of the American Chemical Society*, 2012, **134**, 7344-7350.
4. W. Rao, P. Kothandaraman, C. B. Koh and P. W. H. Chan, *Advanced Synthesis & Catalysis*, 2010, **352**, 2521-2530.
5. E. M. L. Sze, M. J. Koh, Y. M. Tjia, W. Rao and P. W. H. Chan, *Tetrahedron*, 2013, **69**, 5558-5565.
6. J. M. Senedese, F. Rinaldi-Neto, R. A. Furtado, H. D. Nicollela, L. D. R. de Souza, A. B. Ribeiro, L.

S. Ferreira, G. M. Magalhães, I. Z. Carlos and J. J. M. da Silva, *Biomedicine & Pharmacotherapy*, 2019, **111**, 331-337.

7. J. A. Fischer, S. Muller-Weeks and S. J. Caradonna, *Cancer research*, 2006, **66**, 8829-8837.
8. G. Li, J. W. Boyle, C.-N. Ko, W. Zeng, V. K. W. Wong, J.-B. Wan, P. W. H. Chan, D.-L. Ma and C.-H. Leung, *Acta Pharmaceutica Sinica B*, 2019, **9**, 537-544.
9. S. Chung, J. B. Parker, M. Bianchet, L. M. Amzel and J. T. Stivers, *Nature chemical biology*, 2009, **5**, 407.
